# Combined effects of ocean acidification and temperature on larval and juvenile growth, development and swimming performance of European sea bass (*Dicentrarchus labrax*)

**DOI:** 10.1371/journal.pone.0221283

**Published:** 2019-09-06

**Authors:** Louise Cominassi, Marta Moyano, Guy Claireaux, Sarah Howald, Felix C. Mark, José-Luis Zambonino-Infante, Nicolas Le Bayon, Myron A. Peck

**Affiliations:** 1 Institute of Marine Ecosystem and Fisheries Science, Center for Earth System Research and Sustainability (CEN), University of Hamburg, Hamburg, Germany; 2 Université de Bretagne Occidentale, LEMAR (UMR 6539), Centre Ifremer de Bretagne, Plouzané, France; 3 Alfred Wegener Institute Helmholtz Centre for Polar and Marine Research, Integrative Ecophysiology, Bremerhaven, Germany; 4 Ifremer, LEMAR (UMR 6539), Laboratory of Adaptation, Reproduction and Nutrition of Fish, Centre Ifremer de Bretagne, Plouzané, France; Helmholtz-Zentrum fur Ozeanforschung Kiel, GERMANY

## Abstract

Ocean acidification and ocean warming (OAW) are simultaneously occurring and could pose ecological challenges to marine life, particularly early life stages of fish that, although they are internal calcifiers, may have poorly developed acid-base regulation. This study assessed the effect of projected OAW on key fitness traits (growth, development and swimming ability) in European sea bass (*Dicentrarchus labrax*) larvae and juveniles. Starting at 2 days post-hatch (dph), larvae were exposed to one of three levels of *P*CO_2_ (650, 1150, 1700 μatm; pH 8.0, 7.8, 7.6) at either a cold (15°C) or warm (20°C) temperature. Growth rate, development stage and critical swimming speed (U_crit_) were repeatedly measured as sea bass grew from 0.6 to ~10.0 (cold) or ~14.0 (warm) cm body length. Exposure to different levels of *P*CO_2_ had no significant effect on growth, development or U_crit_ of larvae and juveniles. At the warmer temperature, larvae displayed faster growth and deeper bodies. Notochord flexion occurred at 0.8 and 1.2 cm and metamorphosis was completed at an age of ~45 and ~60 days post-hatch for sea bass in the warm and cold treatments, respectively. Swimming performance increased rapidly with larval development but better swimmers were observed in the cold treatment, reflecting a potential trade-off between fast grow and swimming ability. A comparison of the results of this and other studies on marine fish indicates that the effects of OAW on the growth, development and swimming ability of early life stages are species-specific and that generalizing the impacts of climate-driven warming or ocean acidification is not warranted.

## Introduction

Over the last 150 years, the burning of fossil fuels has contributed to an increase in atmospheric CO_2_ from approximately 280 to 410 ppm and a further increase (730 to 1020 ppm) is anticipated by the end of 2100 [1,2]. This increased concentration of atmospheric CO_2_ (*P*CO_2_) has enhanced greenhouse warming around the globe at a rate of ~ 0.2°C per decade in the past 30 years [3] and, after dissolving in the oceans, it is causing ocean acidification (OA). During the 20^th^ century, the pH of ocean surface waters has decreased by 0.1 pH units and a further reduction of 0.3–0.5 pH units is expected to occur by the end of the present century [4]. These changes in *P*CO_2_, pH and temperature of the oceans have impacted the distribution, physiological performance, morphology and behavior of marine organisms [5]. The effect of the interaction between OA and warming (OAW) on marine flora and fauna is difficult to predict, especially since impacts are often life stage- and species-specific, and ocean warming (OW) could either offset [6] or aggravate impacts of OA.

Gaining a mechanistic, physiologically-based understanding of how OAW affects marine flora and fauna is essential for reliable projections of future effects of climate change [7]. Studies examining the consequences of OA on marine organisms have mainly focused on calcifying invertebrates [8, 9] with far fewer studies conducted on fish [10]. In fish, accumulating bicarbonate is a classical response aimed at regulating acid-base balance when the internal milieu acidifies. The net increase of HCO_3_^-^ in plasma occurs in exchange for Cl^-^, predominantly at the gills [11], but this HCO_3_^-^ / Cl^-^ exchange eventually reaches a species-specific threshold beyond which acid-base regulation may occur at the expense of internal ionic/osmotic balance [12]. Juvenile and adult fish have efficient acid-base and osmo-ionic regulatory systems and are particularly tolerant to environmental hypercapnia and acidification [13]. Young larvae, however, have not yet developed full regulatory capacity and, consequently, they are expected to be more sensitive to changes in internal *P*CO_2_ and pH [14, 15]. This appears to be the case as several studies have reported decreased survival and/or abnormal development in marine fish larva exposed to OA [16, 17, 18] and behavior can be impacted via impaired sensory abilities such as olfaction [19]. Within the thermal tolerance window, warming increases rates of biochemical reactions and, thus, overall energy requirements and oxygen demand. After a certain thermal threshold is exceeded, however, deterioration in cellular activities occurs, resulting in reduced tissue and organismal-level performance. Decrements in performance may be due to the limited capacity of the oxygen transport chain to sustain temperature-driven increases in ATP production by mitochondria [20, 21, 22]. It might also be explained by the ‘multiple performances–multiple optima’ (MPMO) hypothesis, which posits that each physiological activity has its own thermal optimum which can shift with life stage and the nature of the thermal challenge [23].

In early life stages of marine fish, growth and development lead to improvements in locomotor performance, a fundamental trait that influences food acquisition, predator avoidance and habitat connectivity (e.g. between spawning sites and larval nursery areas) [24]. Hence, locomotor performance is a key determinant of Darwinian fitness. Critical swimming speed (U_crit_; [25]) is a popular measure of swimming performance, estimating the athleticism of fish. The U_crit_ is also a well-established index to monitor the ontogeny of swimming performance in marine fish larvae, especially in tropical species [26]. Moreover, U_crit_ has been frequently used to evaluate the effects of environmental factors (e.g. temperature, dissolved oxygen concentration, presence of toxins and pathogens) on the physiological performance of fish [27]. Therefore, when examining the effects of OA and/or OW on organismal-level performance, examining U_crit_, along with growth and development, provides an integrated measure of physiological impact with clear ecological relevance.

The European sea bass (*Dicentrarchus labrax*) is one of the most important commercial and recreational fish species in the Northeast (NE) Atlantic and Mediterranean Sea, and potential sensitivity to stressors can negatively impact the productivity of this species and its fisheries [28]. Due to its importance as an aquaculture target, standard rearing protocols exist for rearing sea bass early life stages, and swimming performance has been well measured, including inter-individual variability and repeatability [29, 30]. We examined the effects of OAW on the somatic growth, development and swimming capacity throughout the larval and early juvenile phase of sea bass reared at two temperatures (15°C and 20°C) and three *P*CO_2_ levels (650, 1150, 1700 μatm; pH 8.0, 7.8, 7.6). We compared our results with previous studies conducted on sea bass and critically reviewed the literature published on the effects of OA, OW, and OAW on the swimming ability of marine fish early life stage.

## Materials and methods

The present work was performed within Ifremer-Centre de Bretagne facilities (agreement number: B29-212-05). Experiments were conducted according to the ethics and guideline of the French law and approved by the governmental ethics committee of the Brittany region (Comité d'Ethique Finistérien en Experimentation Animal, CEFEA, registering code C2EA-74) (Authorization APAFIS 4341.03).

### Animals and experimental conditions

#### Water parameters

The larvae and post-larval juveniles were incubated within 6 different OAW treatments. The acidification conditions included three different CO_2_ partial pressures (*P*CO_2_). For the control treatment, the targeted level of CO_2_ was set to approximately 650 μatm, today’s ambient situation in coastal waters of Brittany [31, 32], with an annual mean *P*CO_2_ level of 603 μatm (range 284–888 μatm) in the Bay of Brest, in 2014 [33]. Climate projections indicate that the oceans will reach about 1000 μatm *P*CO_2_ in the next 130 years [2, 4, 34]. A second treatment was based on the IPCC Representative Concentration Pathway (RCP) 8.5 scenario projecting a Δ*P*CO_2_ of ~500 μatm above current values (labelled Δ500, approx. 1150 μatm) [2]. Sea bass juveniles and adults are usually found in coastal waters and estuaries where the impact of *P*CO_2_ might be exacerbated [35, 36]. Wallace et al., 2014 [37] reported *P*CO_2_ values >2000 μatm in northeast US estuaries, while values up to 3000 μatm were recorded in coastal areas of the SW Baltic Sea [38]. Based on those data, and *P*CO_2_ values of European estuaries provided by Frankignoulle et al., 1998 [39], a higher CO_2_ treatment of Δ1000 μatm from ambient level (labelled Δ1000, approx. 1700 μatm) was applied representing CO_2_ condition encountered occasionally by the adults and may become more common under future climate. Acidification conditions were crossed with two temperatures: a ‘cold’ (ambient condition) and a ‘warm’ (global warming) treatment. Under ambient condition, larvae were reared at 15°C and juveniles experienced 15 to 18°C (natural, seasonal differences reflecting ambient summer conditions in the Bay of Brest (see http://marc.ifremer.fr/en/results/temperature_and_salinity/mars3d_channel_bay_of_biscay_model/(typevisu)/map/(zoneid)/sudbzh#appTop) [40, 41]. In the warm treatment, larvae were reared at 20°C and juveniles experienced 20 to 23°C (5°C warmer than the cold treatment). The 5°C increase was based on the ‘business-as-usual (RCP 8.5) scenario predicted by the general circulation models (GCMs) by the end of the century [42]. We applied constant temperatures for larvae and seasonally changing temperatures for juveniles to better depict thermal conditions experienced by these life stages in the wild. Due to relatively fast rates of growth and development, sea bass larvae experience less differences in temperature compared to juveniles. Larvae hatch offshore, and then juveniles enter estuaries in the late spring and grow in these waters through the summer months [43, 44].

The sea water was pumped from a depth of 20 m approximately 500 m from the coastline in the Bay of Brest, passed through a sand filter (~500 μm), heated (tungsten, Plate Heat Exchanger, Vicarb, Sweden), degassed using a column, filtered using a 2 μm membrane and finally UV sterilized (PZ50, 75W, Ocene, France) assuring high water quality. Replicate treatment tanks (n = 3 for larval rearing and n = 2 for juvenile rearing) were supplied with sea water via header tanks where water *P*CO_2_ was controlled using an IKS Aquastar system (IKS Computer Systeme GmbH, Germany). This design used interdependent treatment replicates which was corrected using tank as a random factor in the analysis [45]. The system continuously measured water pH and controlled a solenoid valve connected to a CO_2_ cylinder. This valve controlled the amount of CO_2_ injected into the header tank water, which supplied the fish rearing tank. Temperature and pH were checked daily with a WTW 3110 pH meter (Xylem Analytics Germany, Weilheim, Germany; with electrode: WTW Sentix 41, NBS scale) before feeding the fish. The pH meter as well as the IKS Aquastar system were calibrated daily with NBS certified WTW technical buffers pH 4.01 and pH 7.00 (Xylem Analytics Germany, Weilheim, Germany). Total alkalinity was measured once a week following the protocol of Anderson and Robinson, 1946 [46], and Strickland and Parsons, 1972 [47]: a 50 ml sample of filtered tank water (200 μm nylon mesh) was mixed with 15 ml HCl (0.01 M) and pH was measured immediately. Total alkalinity was then calculated with the following formula:

$$TA=\frac{V_{HCl}∙c_{HCl}}{V_{sample}}-\frac{\left(V_{HCl}+V_{sample}\right)}{V_{sample}}∙\frac{\left\{H^{+}\right\}}{\gamma_{H^{+}}}\;[\frac{mol}{l}]$$

With: TA—total alkalinity [mol * l^-1^], V_HCl_—volume HCl [l], c_HCl_—concentration HCl [mol * l^-1^], V_sample_—volume of sample [l], H^+^—hydrogen activity (10^-pH^), γ^H+^—hydrogen activity coefficient (here γ^H+^ = 0.758).

The Microsoft Excel macro CO2sys [48] was used to calculate seawater carbonate chemistry, the constants after Mehrbach et al., 1973 [49] (as cited in CO2sys) refit by Dickson et al., 1987 [50] (as cited in CO2sys), were employed. Values of pH are presented on the free proton concentration scale (pH_free_) [51]. Oxygen saturation (WTW Oxi 340, Xylem Analytics Germany, Weilheim, Germany) and salinity (WTW LF325, Xylem Analytics Germany, Weilheim, Germany) were measured once a week together with total alkalinity, from juvenile stage onwards (Table 1).

**Table 1:** Water parameters during the larval (L) and juvenile (J) rearing done in this study. Larval period from 21.01.2016 (3 days post-hatch (dph)) until 04.03.2016 (46 dph) and 18.03.2016 (60 dph) for warm (W) and cold (C) life condition respectively; juvenile period until 24.10.2016 (280 dph) and 08.02.2017 (387 dph) for warm (W) and cold (C) life condition respectively. Values show mean ± SE overall replicate tanks per condition. Temperature (Temp.) and pH (free scale) were measured daily; salinity and total alkalinity (TA) and oxygen weekly; *P*CO_2_ was calculated with CO2sys. Inflow sea water (SW) parameters were measured in 2017 and 2018 and annual average values are shown. A, Ambient *P*CO_2_; Δ500, ambient + 500 μatm CO_2_; Δ1000, ambient + 1000 μatm CO_2_.

Treatment	pH_Free_ (-)	Temp. (°C)	Salinity (psu)	O2 (% airsat.)	TA ()	*P*CO_2_ (μatm)
L C A	7.95±0.01	15.3±0.0	33.0±0.1	-	2364±17	656±16
L C Δ500	7.77±0.01	15.3±0.0	33.0±0.1	-	2382±19	1041±26
L C Δ1000	7.58±0.00	15.3±0.0	33.0±0.1	-	2394±26	1682±26
L W A	7.88±0.01	20.0±0.1	33.1±0.1	-	2369±21	832±13
L W Δ500	7.79±0.01	20.0±0.1	33.1±0.1	-	2383±22	1057±30
L W Δ1000	7.60±0.01	20.0±0.1	33.1±0.1	-	2380±23	1672±33
J C A	7.97±0.01	16.0±0.2	34.2±0.1	90.9±0.5	2396±18	655±18
J C Δ500	7.75±0.01	16.0±0.2	34.2±0.1	92.2±0.6	2404±19	1107±21
J C Δ1000	7.55±0.01	16.1±0.2	34.2±0.1	90.9±0.6	2399±19	1841±40
J W A	7.92±0.01	21.9±0.2	35.0±0.2	90.2±0.9	2418±12	788±22
J W Δ500	7.78±0.01	21.8±0.2	35.0±0.2	90.5±0.7	2420±15	1133±43
J W Δ1000	7.59±0.01	21.9±0.2	35.0±0.2	91.3±0.6	2423±12	1808±65
SW cold	8.05±0.01	14.5±0.5	33.0±0.2	101.2±0.6	2434±21	522±18
SW warm	7.95±0.02	21.2±0.4	32.7±0.1	102.3±1.4	2433±28	723±33

#### Animals

Larvae were obtained from an aquaculture facility (Aquastream, Ploemeur-Lorient, France) at 2 days post-hatch (dph) (20.01.2016). Brood stock fish were caught in the sea off Morbihan, France. Four females (mean weight 4.5 kg) were crossed with ten males (mean weight 2.4 kg) which spawned naturally using photothermal manipulation. Conditions in the aquaculture facility during breeding were as followed: 8h45 light and 15h15 darkness, 13°C, 35 psu, pH 7.6. Spawning of eggs took place on 15.01.2016; larvae hatched on 18.01.2016 and were transported to our laboratory facilities on 20.01.2016.

Larval rearing was performed in a temperature-controlled room using black, 35-L tanks initially stocked with *ca*. 5000 larvae tank^-1^ in order to accommodate our sampling design. Allocation among experimental tanks took place at 3 dph (21.01.2016). During the following three days, the temperature for the warm condition was increased 1°C during the first day and 2°C during each of the following days. The *P*CO_2_ conditions were applied directly after fish allocation to the experimental treatments. Starting at 7 dph (mouth opening), larvae were fed with live brine shrimp (*Artemia salina)* nauplii, hatched from High HUFA Premium cysts (Catvis, AE's-Hertogenbosch, Netherlands). From 7 to 16 dph a concentration of ~120 nauplii per larva day^-1^ was delivered, after 16 dph concentration was ~800 nauplii per larva day^-1^. Until 33 dph, larvae were fed newly hatched (24-h old) nauplii. Older larvae were fed with nauplii enriched with cod liver oil and dry yeast for 24 h. The nauplii were transferred from their storage tanks (one per temperature condition) to the larval rearing tanks using peristaltic pumps at ad libitum feeding concentrations continuously during 6 hours. Larvae experienced a 15-h photoperiod (7:00 to 22:00), the light intensity progressively increased with larval age, from total darkness to 96 lux according to S1 Table. Median larval mortality (n = 18 tanks) was 30%, see S2 Table. Flow rate through the larval tanks was 0.18 L min^-1^, corresponding to a water exchange of 30% per hour, and organic compounds were removed from the tanks using a protein skimmer.

Fish were moved from the larval to juvenile tanks at 50 dph and 65 dph for fish reared in the warm and cold life conditions, respectively. Fish were counted, and all individuals from replicate tanks at one condition (temperature x *P*CO_2_) were pooled in the same tank for about three weeks and then randomly allocated to two 670-L treatment tanks (S1 Fig). Having only two replicates limited our ability to estimate variance but dividing the fish randomly removed any potential effect of larval rearing tank. Mortality of 24.8 to 43.4% occurred after loading to the juvenile tanks, likely due to handling stress (S3 Table). Juveniles were fed daily with commercial fish food (Neo Start) (Le Gouessant, Lamballe, France). Food was distributed ad libitum via automatic feeders. Size and amount was adjusted all through the juvenile rearing period, as recommended by the supplier. This amount was calculated according to the tank biomass, number of fish and temperature. Around 200 dph, for example, 90 g tank^-1^ day^-1^ and 160 g tank^-1^ day^-1^ of food pellets was distributed for the 15°C-reared fish (mean biomass ~ 6.9 kg) and the 20°C-reared fish (mean biomass ~ 4.2 kg), respectively. Photoperiod was adjusted each week to mimic natural conditions. Uneaten food and feces were siphoned from tanks each day (after pH measurements). Water flow rates maintained oxygen saturation levels above 90%.

### Swimming tests

#### Larvae

Swimming tests and morphological measurements were performed from 15 dph until the end of the larval stage i.e., when the caudal fin was completely formed. Swimming experiments were conducted on larvae from the six treatments (*P*CO_2_ x T) conditions. In each swimming trial, 3 randomly selected larvae from each replicate tank (9 larvae per treatment) were measured. Trials were conducted every 3 to 5 days, and 6 to 7 trials were conducted per treatment.

All measurements of U_crit_ were performed on an individual larva swimming in one lane (24 x 3 x 2.5 cm) of a custom-made Brett-type flume [52]. Water was pumped (universal, EHEIM, Germany) from a header tank into the flume and velocity was adjusted using a valve calibrated to water flow rate. A laminar flow was made by passing the water through a honeycomb section (length = 10 cm) placed upstream and a mesh screen was located at the downstream end of the lane. Pilot trials with dye ensured that cross-sectional water velocity in the lane was homogenous. Treatment water conditions of the tested larva were maintained in the flume. Temperature was controlled using a cooling/heating system (Tr10, TECO, Italy) and *P*CO_2_ was maintained by injecting CO_2_ directly in the water of the header tank via a gas diffuser.

A larva was introduced into the swimming lane and was acclimated to the lowest water velocity for 5 min (Table 2). The water velocity was then increased at a rate of 0.5 BL s^-1^ every 3 min until the larva was unable to swim against the flow and drifted to the downstream mesh screen. At the beginning of each trial, the average length of the fish in each replicate tank was determined so that a standard velocity increment could be established (among all trials). Larvae swam in the middle of the chamber suggesting minimal or no wall effects. Once the test was completed, the larva was euthanized with an overdose of anesthetic (Tricaine methane-sulfonate MS222, PharmaQ Limited, Hampshire, United Kingdom, as prescribe by the European legislation to minimize fish stress), digitally photographed under a stereomicroscope (Leica MZ 16, Wetzlar, Germany) and stored in 4% formalin. Body length (BL), body height (BH) and tail flexion angle, were measured using ImageJ [53]. For preflexion larvae, BL was equal to the notochord length, which corresponded to the length from the tip of the snout to the end of the notochord. For flexion and postflexion larvae, BL was determined by measuring standard length, corresponding to the distance from the tip of the snout to the posterior end of the hypural plate. The notochord angle was also measured to estimate the size at which larvae reached the postflexion stage. Unfortunately, due to the small sample size of larvae < 10 mm in length (n = 45 for 15°C and n = 54 for 20°C), it was not possible to run a logistic regression to calculate the mean larval size at which 50% of the larvae completed flexion. Instead, we determined the size at which postflexion was first observed.

**Table 2:** Summary of the methodology used during the critical swimming trials with larval (L) and juvenile (J) European sea bass. Abbreviations: BL, body length; C, cold treatment; W, warm treatment; A, Ambient *P*CO_2_; Δ500, ambient + 500 μatm CO_2_; Δ1000, ambient + 1000 μatm CO_2_.

Treatment	BL (mm)	Age (dph)	Total larvae tested (n)	Acclimation period	Water flow steps (cm s^-1^)	Time steps (min)
L C A	6.67–16.69	21–59	58	5 min at 0.8–1.6 cm s^-1^	0.4–0.8 (0.5 Bls)	3
L C Δ500	7.45–16.83	21–58	51			
L C Δ1000	7.82–17.97	22–58	59			
L W A	7.55–16.66	17–46	54	5 min at 0.8–1.6 cm s^-1^	0.4–0.8 (0.5 Bls)	3
L W Δ500	7.61–17.41	18–45	55			
L W Δ1000	6.35–17.35	18–45	54			
J C A	83.2±7.1	239–242	30	30 min at 7 cm s^-1^	2.8	10
J C Δ500			30			
J W A	119.9±9.3	233–238	30	30 min at 7 cm s^-1^	5.6	10
J W Δ500			30			

#### Juveniles

Juveniles from four treatments were tested: cold and warm temperatures at both ambient *P*CO_2_ (650 μatm) and RCP8.5 (Δ1000; 1700 μatm). When tested, juveniles in the cold and warm treatments were 242 and 233 dph and had a mean (± s.e.m) BL of 83.2(0.8) and 119.9(1.2) mm, respectively. For each treatment, thirty individuals (~15 per replicate tank) were tested. For each run, fish were tested in groups of 8 or 5 individuals, for 15°C and 20°C- reared fish, swimming in a 46 x 14 x 14 cm chamber of the Loligo Systems swim tunnel (Denmark). A streamline and homogenous flow was maintained using honeycomb section (confirmed by dye tests). Fish were tested at their treatment water conditions (see section 2.2.1). Water velocity was calibrated with a vane-wheelflow meter (HFA, Höntzsch GmbH, Germany), and controlled by an AC motor [54]. Acclimation time was 30 min (Table 2).

Fish were considered to be exhausted when they were up against the downstream grid for 5 consecutive seconds. Without interrupting the flow, these individuals were then removed from the tunnel, via a hatch located above the grid, anesthetized, measured (BL) and transferred to a recovery tank before being returned to their rearing tank where they were maintained for future research. For juveniles, BL is equivalent to standard length. The corresponding time and water velocity was recorded. The test was completed when all 5 fish were removed from the swim chamber.

#### U_crit_ measurement

The U_crit_ (cm s^-1^) was calculated using the equation provided by Brett, 1964 [25], which adds the velocity of the most recently completed increment to the product of the incremental increase in velocity and the proportion of the final increment completed before fatigue. No correction for the solid blocking effect of the fish was considered, as the total cross-sectional area of the fish did not exceed 5% of that of the swimming chamber [55] (Table 2). Larvae which did not orientate themselves to start swimming during the acclimation period were removed from the test and dataset.

### Statistical analysis

Differences in growth (in BL) across treatments were analyzed, in larvae, with a linear mixed model that included fixed effects (age, temperature, *P*CO_2_) and random effects (tank). Similarly, differences in BH were also tested with a linear mixed model (BL, temperature and *P*CO_2_ as fixed effects, tank as a random effect). When no effect of OA was observed, data were pooled across OA treatments and regressions were calculated on pooled data. In larvae, inter-individual variability in U_crit_ was large and increased with body size, thus, we used quantile regression to estimate the maximum U_crit_–at-size (i.e. maximum swimming capacity) across treatments [56]. A backward model selection procedure was used to identify variables (e.g. size, temperature, *P*CO_2_) influencing maximum U_crit_ starting with the most complex model (including all interactive effects among fixed factors) and ending with only significant factors. Models were fit to the upper 85 to 95% quantiles (in 1% steps) to ensure patterns were consistent, and treatment differences were tested with an ANOVA. Model residuals were tested for a potential effect of rearing tank with an ANOVA. All quantile regression analyses were done with the “quantreg” package in R. Normality and homoscedasticity of data were tested using Shapiro-Wilk and Levene tests, respectively. The effects of temperature and *P*CO_2_ levels on the percentage (%) of larvae with the ability / choice to swim was tested using two-way ANOVAs after logit transformation of the data. Larvae not swimming already at acclimation speed were excluded from the other analysis. The effect of *P*CO_2_ on juvenile U_crit_ was tested using one-way ANOVA when assumption of normality (Shapiro-Wilk) and homoscedasticity (Levene test) were met (case for 20°C-reared fish after log10 transformation). ANOVA included *P*CO_2_ as a fixed effect and run as a random effects. When one or more of these assumptions was not met (for 15°C-reared fish), a generalized linear mixed-effects model (GLMM) was performed, including fixed effect (*P*CO_2_) and random effects (run). All statistical analyses were performed using R (version 3.4.1, R Core Team 2014).

## Results

### Larval growth and development

**Fig 1:**
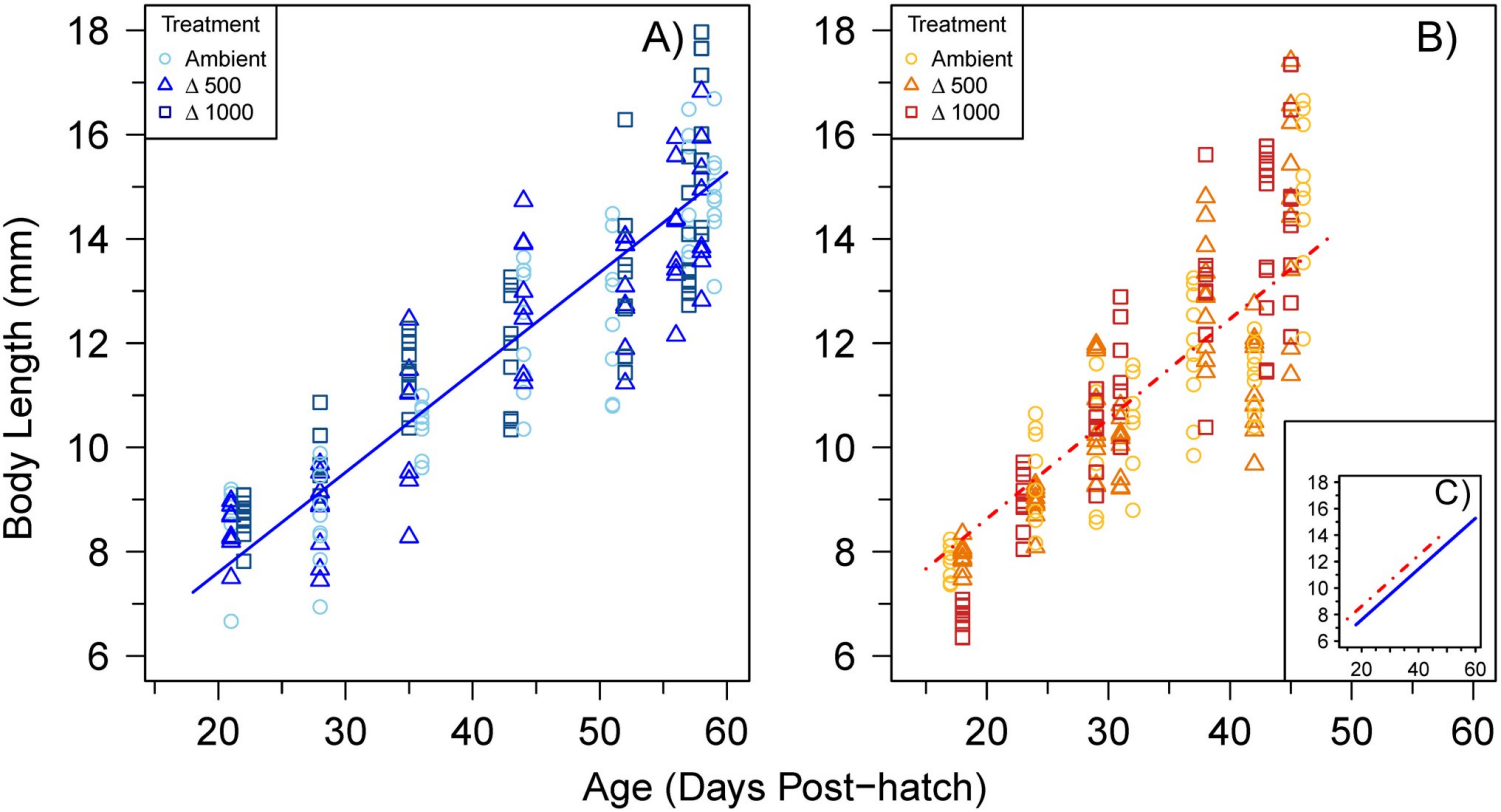
Body length (BL) with age, in days post-hatch, of European sea bass larvae reared at A) cold condition (15°C) and B) warm condition (20°C). Symbols and colors indicate the *P*CO_2_ treatment (A, Ambient *P*CO_2_; Δ500, ambient + 500 μatm CO_2_; Δ1000, ambient + 1000 μatm CO_2_). Regression (mean ± SE parameter estimates) are included: 15°C, (n = 180) BL = 017(0.01)*Age + 4.62(0.28), R^2^ = 0.80, p < 0.001; 20°C (n = 190) BL = 0.23(0.01)*Age + 3.44(0.36), R^2^ = 0.73, p < 0.001). For clarity, both regression lines are compared in subpanel **C**) (insert).

**Fig 2:**
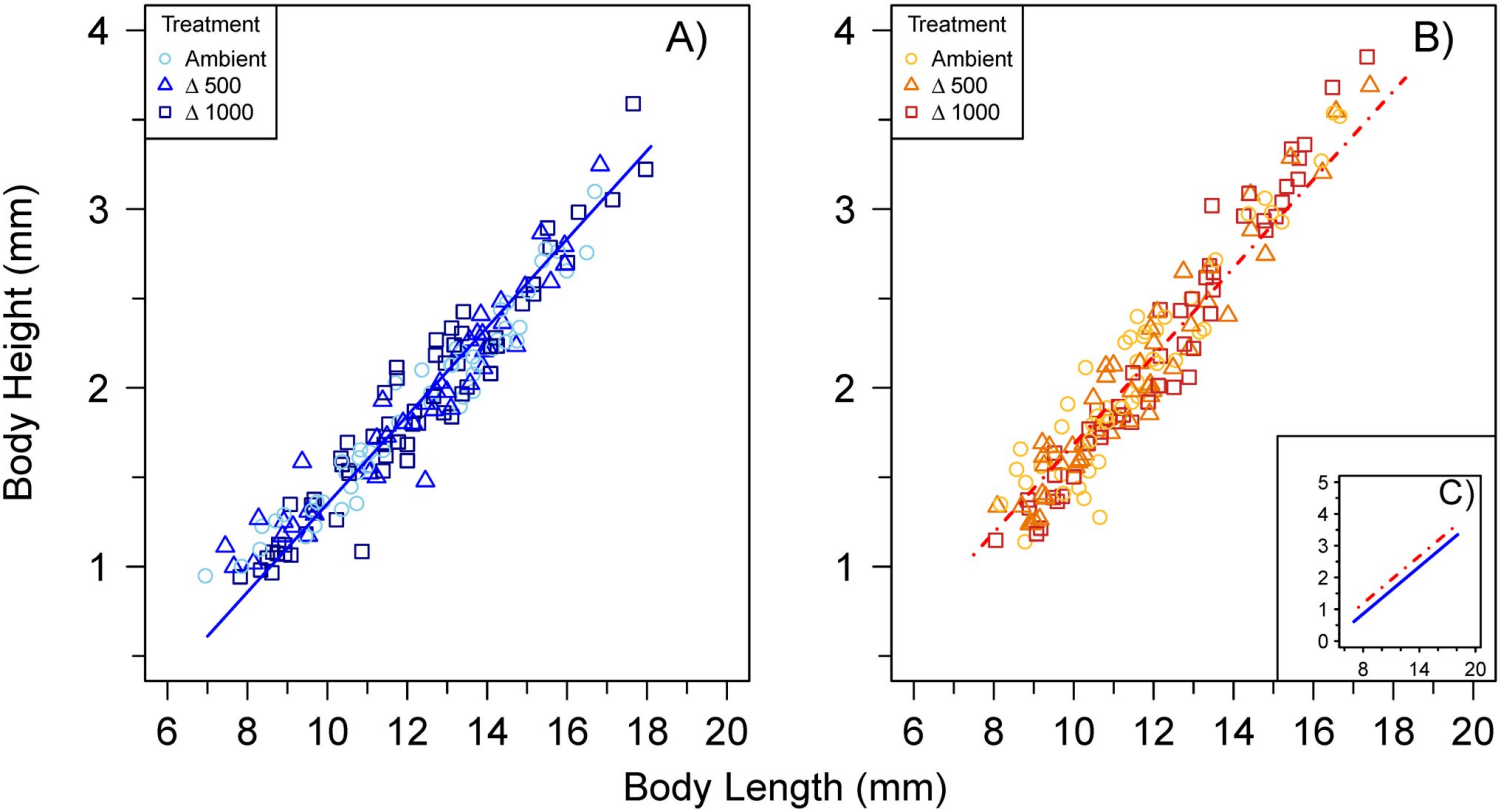
Body height (BH) versus body length (BL) of European sea bass larvae reared at A) cold condition (15°C) and B) warm condition (20°C). Symbols and colors indicate the *P*CO_2_ treatment (A, Ambient *P*CO_2_; Δ500, ambient + 500 μatm CO2; Δ1000, ambient + 1000 μatm CO2). Regression equations with mean (± SE) parameter estimates are: 15°C, (n = 160) BH = 0.22(0.00)*BL—0.83(0.06), R^2^ = 0.93, p < 0.001; 20°C (n = 190) BH = 0.28(0.01)*BL -1.14(0.07), R^2^ = 0.93, p < 0.001). For clarity, both regression lines are compared in subpanel **C**) (insert).

The mean (± SE) growth rate of sea bass larvae at ambient, Δ500, Δ1000 (650, 1150, 1700 μatm *P*CO_2_; pH 8.0, 7.8 and 7.6) was, 0.17 (0.01), 0.17 (0.01) and 0.16 (0.01) mm d^-1^, respectively, at 15°C and 0.21 (0.02), 0.21 (0.02) and 0.28 (0.03) mm d^-1^, respectively, at 20°C. Larval growth rate was significantly higher at 20°C compared to 15°C (p < 0.001), but there was no significant effect of *P*CO_2_ treatment (p = 0.120) (Fig 1). Larval BH increased linearly with BL (Fig 2) and this relationship was significantly impacted by temperature (p< 0.001) but not *P*CO_2_ (p = 0.805). For example, 10-mm larvae at 20°C had a 16% larger BH than those reared at 15°C (mean BH of 1.63 and 1.37 mm, respectively). These morphological differences were related to a faster development at the warmer temperature. At 15°C, notochord flexion was completed between 9.0(±0.3; mean±SE) and 10.8(±0.1), 8.7(±0.2) and 11.1(±0.5), and 9.8(±0.2) and 11.4(±0.2) mm BL at ambient, Δ500, and Δ1000 *P*CO_2_, respectively. At 20°C, notochord flexion was completed between 7.8(±0.1) and 9.6(±0.3), 7.9(±0.1) and 8.9(±0.1), and 7.0(±0.2) and 9.4(±0.1) mm BL in the ambient, Δ500 and Δ1000 *P*CO_2_ treatments, respectively. Unfortunately, the sample size of <12 mm BL larvae was too small to conduct further analyses.

### Swimming capacity of larvae

**Fig 3:**
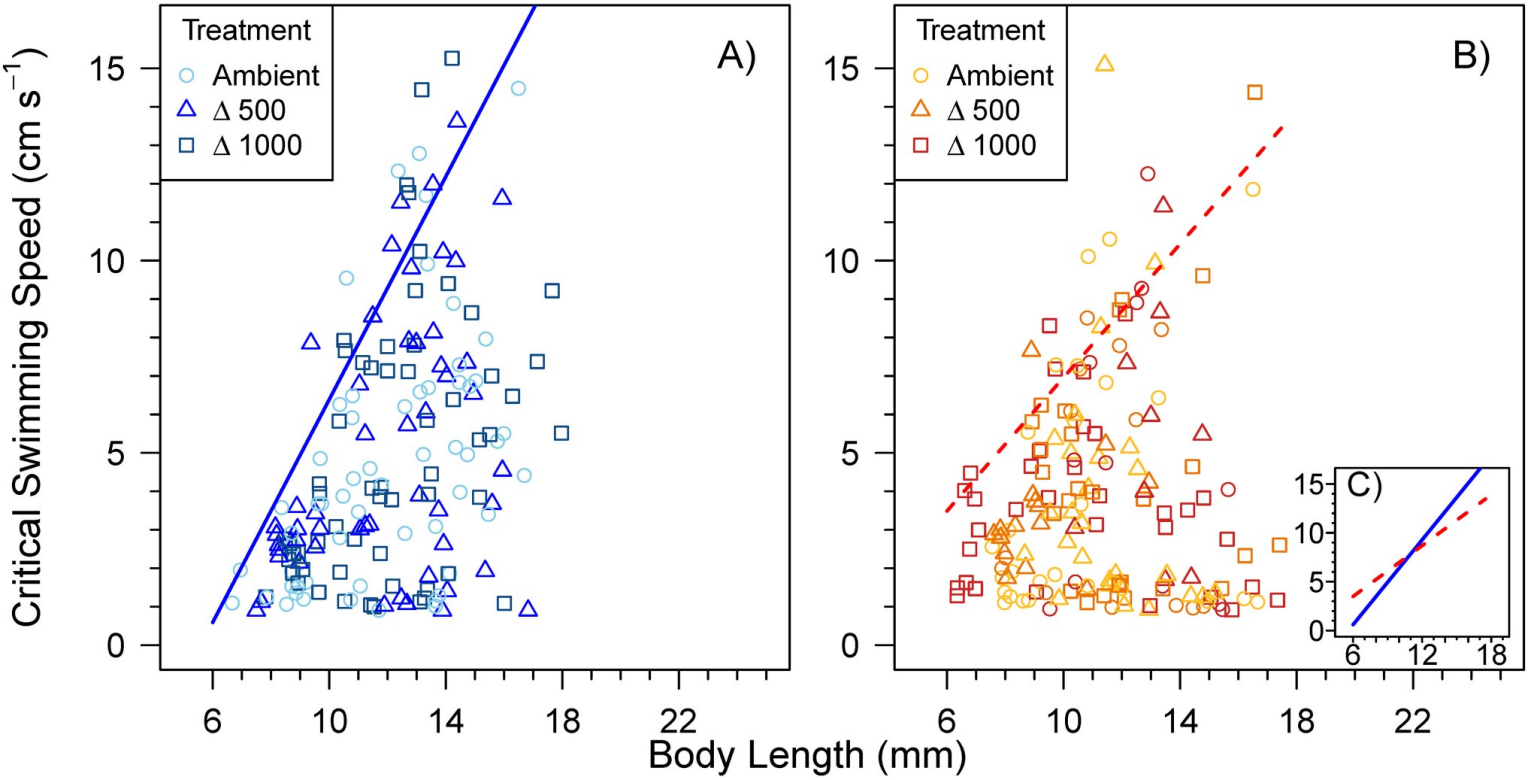
Ontogeny of critical swimming speed (U_crit_, cm s ^-1^) in larvae of European sea bass reared at A) cold condition (15°C; n = 168) and B) warm condition (20°C; n = 163). Symbols and colors indicate *P*CO_2_ levels treatment (A, Ambient *P*CO_2_; Δ500, ambient + 500 μatm CO_2_; Δ1000, ambient + 1000 μatm CO_2_). The solid blue (cold) and dashed red (warm) lines shows the maximum U_crit_ as defined by the 90^th^ percentile (see text). For clarity, both lines are compared (see insert panel **C**).

The U_crit_ and inter-individual differences in U_crit_ of sea bass larvae increased with increasing BL (Fig 3). The final quantile regression model (90^th^ percentile) reported significant effects of BL, temperature and their interaction (S4 Table), but no significant effect of *P*CO_2_ (ANOVA; p = 0.94). There was no significant tank effect on the final model (ANOVA, p = 0.402). The U_crit_ increased faster with BL in cold (15°C) versus warm (20°C) larvae and, at the end of the larval stage (16 mm BL) was 15.1 and 12.2 cm s^-1^ at 15 and 20°C, respectively.

**Fig 4:**
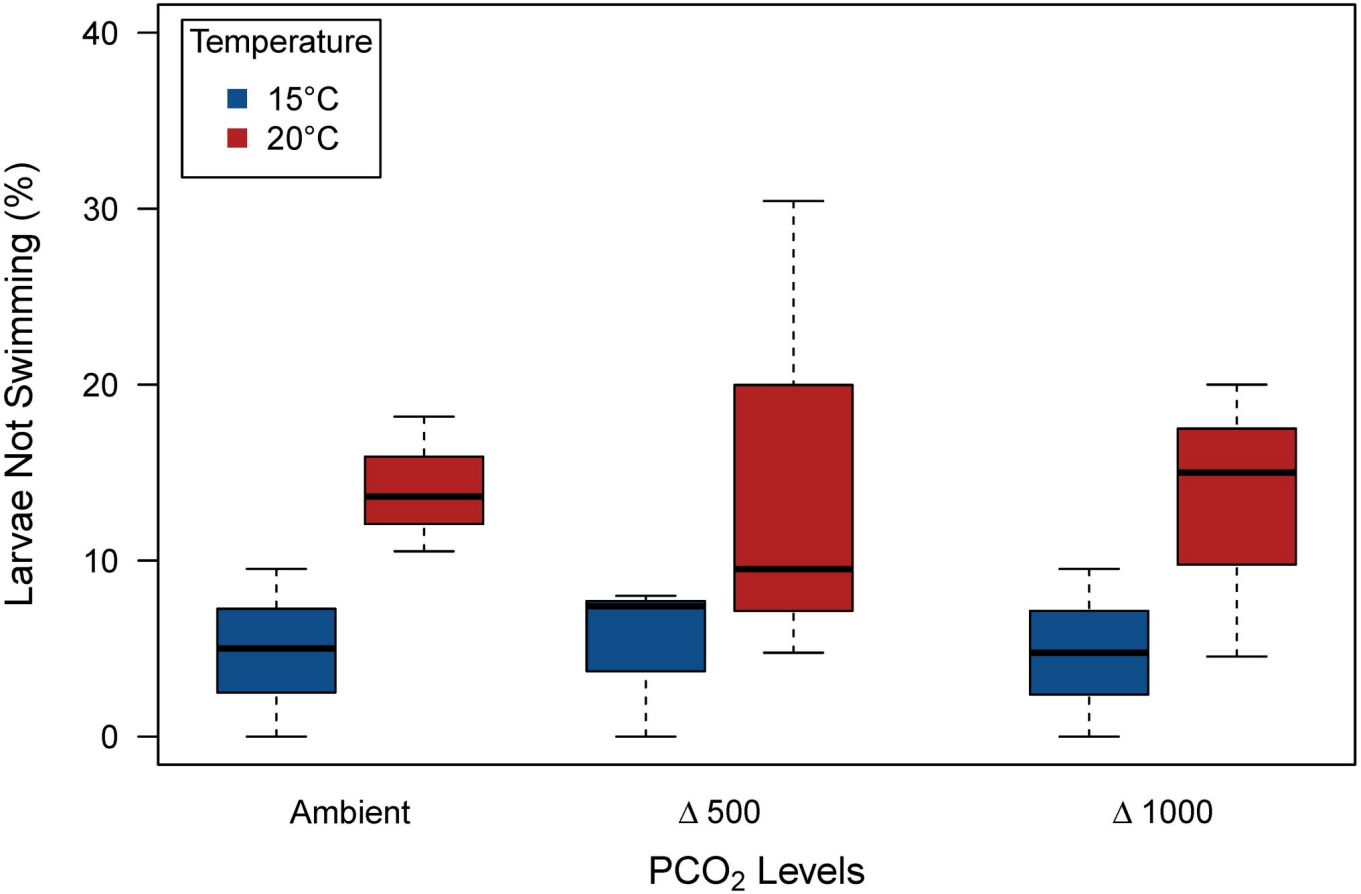
Proportion (in %) of European sea bass larvae not swimming during the U_crit_ trial in the cold (15°C) and warm (20°C) treatments at three *P*CO_2_ levels (Ambient *P*CO_2_; Δ500 = ambient + 500 μatm CO_2_; Δ1000, ambient + 1000 μatm CO_2_).

In the 15°C treatment, the coefficient of variation (CV) of U_crit_ increased from 35.6 to 57.2% between notochord flexion (BL = 9.0 ± 0.2 mm) and post-flexion (BL = 12.9 ± 0.3 mm) stages. At 20°C, the same life stages (BL = 9.1 ± 0.1 and 12.6 ± 0.2, respectively) had CVs of 46.9 to 73.1%. Some larvae were not able (or chose not) to swim beyond the minimum water velocity used during the acclimation period (0.8 cm s^-1^ for 5 min). The percentage of larvae not swimming was significantly higher at 20°C compared to 15°C (ANOVA, p = 0.007, Fig 4).

### Swimming capacity of juveniles

**Fig 5:**
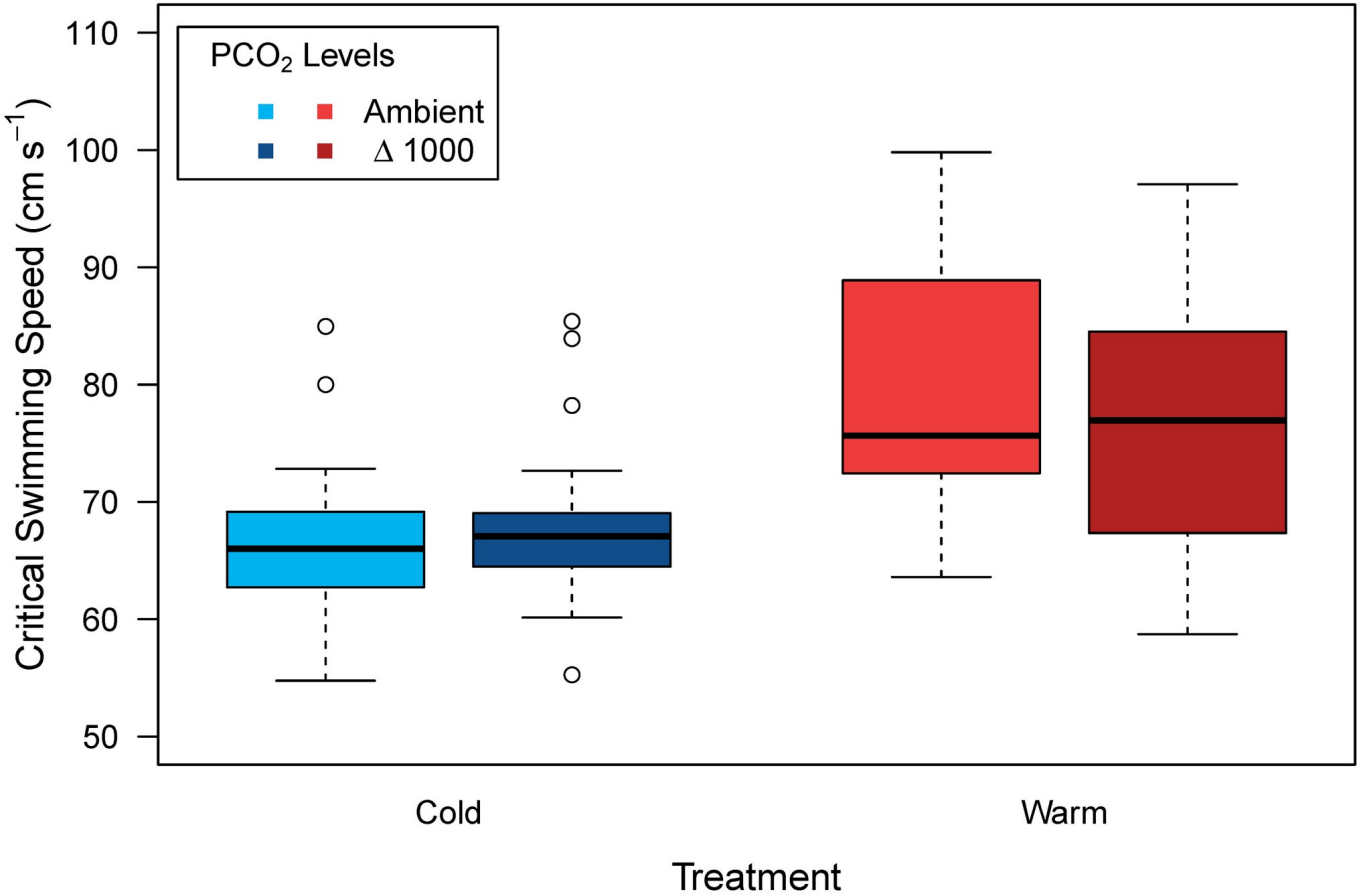
Critical swimming speed (U_crit_, cm s^-1^) in 233 to 242 day post-hatch juvenile European sea bass in the cold (15°C) and warm (20°C) treatments at two *P*CO_2_ levels: Ambient *P*CO_2_ (n = 40 and n = 30, for cold and warm treatment, respectively) and Δ1000, ambient + 1000 μatm CO_2_) (n = 33 and n = 30, for cold and warm treatment, respectively). The raw data are shown in S2 Fig.

The mean (± SE) U_crit_ of juveniles reared in cold and warm conditions was 66.7 (0.7) and 79.2 (1.5) cm s^-1^, respectively. There was no significant effect of *P*CO_2_ treatment on U_crit_ measured in cold (GLMM, p = 0.562) and warm conditions, respectively (ANOVA, p = 0.518). Although a group of 5 fish was tested in each trial, individuals became exhausted at different water velocities and our protocol allowed us to collect individual-level data. Large inter-individual differences were observed in U_crit_, particularly among juveniles reared at warmer temperatures. Body sizes of fish tested in the cold and warm conditions differed, precluding direct comparison between temperatures (Fig 5, S2 Fig).

## Discussion

Recent studies have examined the impact of OA and OAW on early life stage of teleost growth and survival [16, 17]. Large gaps in knowledge, however, persist, particularly on how long-term exposure to OAW may influence the performance of fish larvae and young juveniles. We investigated the effect of OAW on growth, development and swimming ability of European sea bass throughout larval and early juvenile ontogeny. Our results suggest that temperature, but not elevated CO_2_, influenced growth, development and swimming performance. Our results also revealed a trade-off between swimming capacity and fast growth at warm temperatures, casting new light on the determinants of larval survival, dispersal, settlement pattern and recruitment [57, 58, 59].

### Effect of ocean acidification

Juvenile and adult European sea bass inhabit shallow estuaries where physicochemical parameters such as temperature and *P*CO_2_ levels strongly fluctuate over short (diel) and longer (seasonal) time scales [60]. Thus, these life stages experience substantially more variation in water *P*CO_2_ (e.g. daily variation up to 1 pH unit [61]) compared to early life stages. Measurements made between 1992 and 2004 within inner estuaries of the Loire and Gironde Rivers were 452 to 2780 μatm and 612 to 2829 μatm, respectively [62]. On the other hand, sea bass larvae develop in offshore waters where *P*CO_2_ levels are generally stable (annual variation of <0.1 pH units [63]). Taking this into account, it was expected that increased levels of *P*CO_2_ might negatively affect the growth and/or swimming ability of larvae due to poorly developed acid-base regulation and the need to partition energy between homeostasis-related mechanisms and ecologically important activities.

Contrary to our expectations, the results of the present study indicated that life-long exposure to OA had no significant effect on the somatic growth rate and swimming capacity (U_crit_) of sea bass larvae. A previous study on sea bass larvae, using similar exposure, rearing protocols and OA levels (Ambient, Δ500 and Δ1000 (*P*CO_2_ = 650, 1150 and 1700 μatm)), also found no effect of OA on somatic growth at 19°C [49]. These findings are in accordance with those reported in a recent meta-analysis conducted by Cattano et al., 2018 [64], highlighting no overall effects of high CO_2_ on growth. A few studies, however, have reported impacts of OA on growth. For example, OA was associated with a higher growth rate in the clown anemonefish (*Amphiprion percula*) larvae [65] but slower growth rates were reported for gilthead seabream (*Sparus aurata*) [66] and decreased length-at-hatch for inland silversides (*Menidia beryllina*) [16].

Similar to our findings for growth rate, we also found no significant effect of OA on swimming capacity in sea bass larvae and juveniles. These results are in line with those in a number of other studies testing routine swimming characteristics [67, 68] or U_crit_ [65, 69, 70] in marine fish larvae exposed to OA (Table 3). For example, Munday et al., 2009b [65] highlighted that swimming speed (U_crit_) of the clown anemonefish (*Amphiprion percula*) larvae was unaffected by future OA scenarios. Similarly, Bignami et al., 2014 [71] did not observe a significant effect of high acidification on the swimming performance (U_crit_ and mean routine swimming speed) of larval cobia (*Rachycentron canadum*) and mahi-mahi (*Coryphaena hippurus*). Although most studies on marine fish early life stages suggest that the physiological attributes related to swimming performance are not substantially impacted by levels of *P*CO_2_ projected for the end of this century, some studies have reported impaired swimming duration and orientation [72] and reduced U_crit_ as well as the average speed in response to a stimuli [73] (Table 3). Indeed, U_crit_ was significantly lower, in juvenile yellowtail kingfish (*Seriola lalandi*) exposed to high (Δ500) *P*CO_2_. This reduction in U_crit_ values may be linked to 1) reduced motivation to swim and/or 2) reduced physiological performance, two traits that may differ according to individual variation [73]. It is worth noting that high inter-individual variability in U_crit_ was observed across all treatments (CVs of 57 and 73% at 15 and 20°C, respectively, for 9 to 13 mm BL). This large inter-individual variability, however, is generally observed in swimming studies in sea bass [74, 75, 76]. One hypothesis is that these large differences in swimming ability (e.g. maximum 10-fold in 15 mm larvae) also reflect variation in other behavioral traits such as boldness or willingness to swim. Using U_crit_ as a performance test allowed us to compare swimming performance among treatments but also highlighted that results may not only reflect physiological limitation but also potentially inter-individual differences in behavior. Our results, together with these studies, highlight the species-specific nature of the responses to OA and the need for continued mechanistic (physiological-based) studies of potential impacts and the importance of publishing of studies that report no significant effects.

**Table 3:** Summary of published studies investigating the impact of ocean warming (OW), ocean acidification (OA) and their combined effect (OAW) on swimming performance of early life stages (larvae, and juveniles) of marine fishes.

Species		Stressor	Measurement	Life stage	T (°C)		*P*CO_2_ (μatm)		Results	Ref
Common name	Scientific name				Cont	Treat	Cont	Treat		
Anemonefish	*Amphiprion melanopus*	OW	U_crit_	L	28	25	ambient	-	Colder T induced slower development of swimming capacity	[81]
		OAW	RSB	J	28.5	30, 31.5	420	530, 960	Elevated T alone reduced food consumption and foraging activity, combined with high *P*CO_2_ these behaviors were increased	[82]
	*Amphiprion percula*	OA	U_crit_	L	30	-	400	550, 750, 1030	No effect	[65]
Cobia	*Rachycentron canadum*	OA	RSB/ U_crit_	L	ambient	-	400	3500, 5400	No effect	[83]
Dolphinfish	*Coryphaena hippurus*	OA	RSB/ U_crit_	L	ambient	-	400	770 to 2100	No effect	[83]
		OA	RSB	L	26	-	457	1671	Swimming duration and vertical orientation frequency decreased with elevated *P*CO_2_	[84]
Yellowtail kingfish	*Seriola lalandi*	OAW	RSB/ U_crit_	J	19.5	21, 25	589.4	462, 538.3, 959.8, 1010.6	Ucrit and escape performances are enhanced by elevated T. High *P*CO_2_ reduced U_crit_ and distance moved after stimuli	[73]
Meagre	*Argyrosomus regius*	OAW	RSB	L	20	24	350	1400	T increased the time spent swimming. Elevated *P*CO_2_ decreased the time spent swimming and lower capture success	[66]
Gilthead seabream	*Sparus aurata*	OAW	RSB	L	18	22	350	1400	Elevated *P*CO2 decreased the time spent swimming and lower capture success	[66]
Black seabream	*Spondyliosoma cantharus*	OA	RSB	L	23.7	-	356.8	777, 2051.5	Elevated *P*CO_2_ decreased velocity and increased erratic swimming behaviors	[85]
Sand smelt	*Atherina presbyter*	OA	RSB	L	15.9	-	537.1	2080.6	No effect on routine swimming speed. Elevated *P*CO_2_ increased the time to acquire shoaling behaviors and decreased laterization	[86]
		OA	U_crit_	L	16.4	-	600	1000, 1800	No effect	[70]
Atlantic herring	*Clupea harengus*	OA	RSB	L	5 to 10	-	370	1800, 4200	No effect	[68]
Atlantic cod	*Gadus morhua*	OW	U_crit_	L	6	10	ambient	-	U_crit_ decreased with elevated T before metamorphosis	[87]
		OA	RSB	L	7.2	-	370	1800, 4200	No effect	[67]
		OA	U_crit_	J	5	-	528	3080, 5792	No effect	[69]
European sea bass	*Dicentrarchus labrax*	OW	U_crit_	L	18	21	ambient	-	Swimming performance declined with temperature.	[30]
		OAW	U_crit_	L, J	15	20	650	1150, 1700	U_crit_ decreased with elevated T in larvae. No effect of *P*CO_2_.	This Study
Shorthorn sculpin	*Myoxocephalus scorpius*	OW	U_crit_	L	3	6	ambient	-	U_crit_ increased with elevated T before metamorphosis	[87]

Abbreviations: T, temperature; U_crit_, critical swimming speed; RSB, routine swimming behavior; L, larvae; J, juveniles; Cont, control; Treat, treatment.

The fish used in the present study were the progeny of wild-caught adults acclimatized and maintained for about 5 years in the Aquastream aquaculture facility. These fish spawned at a pH of 7.6 which corresponds to our highest CO2 treatment. A number of studies have reported that parental exposure to an elevated level of CO2 may decreased the sensitivity of their progeny to OA [77, 78, 79]. In our study, therefore, the absence of differences in growth and swimming capacity observed among the three *P*CO_2_ treatments could be the result of transgenerational plasticity. According to Griffith and Gobler, 2017 [80], however, the exposure of parents to a stressor (such as low pH) can also increase the sensitivity of their offspring to that stressor and that transgenerational plasticity is highly species-specific.

### Effect of temperature

Rearing at a warmer temperature (20 vs 15°C) resulted in shorter larval stage duration and accelerated growth rate. These results agree with previous studies suggesting that sea bass larvae fed ad libitum display increased growth as temperatures increase to 22°C [88, 89]. Temperatures supporting the maximum growth rate in sea bass larvae have not been estimated, but they are close to 22 to 24°C in adults ~35 cm [74].

The impacts of temperature on performance traits of developing larvae can be complex due to the potential for different thermal optima for different physiological activities (e.g. multiple performances—multiple optima (MPMO) hypothesis [23]) and/or changes in thermal tolerance/optima during ontogeny. Increases in U_crit_ with increasing temperature has been observed in the larvae of a variety of marine fish, including both tropical and temperate species, e.g. red and black anemonefish (*Amphiprion melanopus*) [81], shorthorn sculpin (*Myoxocephalus Scorpius*) [8], yellowtail kingfish (*Seriola lalandi*) [73] and Atlantic herring (*Clupea harengus*) [90]. This increased swimming performance was linked to changes in morphology and developmental rates, as well as to a decrease in water viscosity at warmer temperatures, which is especially relevant for cold-temperate species [91]. An increase in U_crit_ with OW, however, is not expected to be universal but to be context- and species-specific [86]. This is the case with sea bass at the two temperatures tested in the present study. Just prior to metamorphosis and for the same body size, larvae reared at 15°C displayed better swimming ability than larvae reared at 20°C.

Thermal optima for performance measures such as U_crit_ may change during ontogeny. In larger juvenile and adult sea bass maximum scope for activity (MO_2_max) is between 22 and 24°C, and coincides with optimal temperature for U_cirt_ [74, 92]. Unfortunately no information is available on thermal performance curves for maximum scope for activity (MO_2_max) nor U_crit_ in sea bass larvae, but this and previous studies suggest that it is below 20°C [30]. Temperature-dependent shifts in swimming ability may reflect temperature-dependent changes in developmental physiology affecting body shape and skeletal structure and/or muscle characteristics [93, 94, 95]. In our study, for the same BL, larvae were more streamlined at 15 compared to 20°C. A streamlined body shape generally enhanced steady swimming while greater BH induced more irregular and more complex locomotor patterns (e.g. drastic changes in velocity or direction, fast-starts or rapid turns) [94], the velocity of the 20°C-reared fish might then be affect by their rounder body shape. In addition, mitochondrial efficiency can decreased already at sublethal warm temperatures due to membrane associated problems (e.g. proton leak), with consequences for energy demanding processes like swimming. It might be that at 20°C, sea bass larvae are already exposed to a sublethal warm temperature likely to affect mitochondrial structure and thus performance. Although, in a companion study [96], mitochondrial capacities for aerobic ATP production of permeabilized heart fibers were increased in juvenile sea bass reared at the warm versus the cold life condition. Juveniles have generally higher thermal optima than larvae, and this would indicate a rearrangement of biochemical pathways during ontogeny towards evolutionary thermal optima (colder for larvae than for juveniles).

### Trade-off between growth and swimming performance

Faster-growing individuals have better chances of survival by spending less time in life stages particularly vulnerable to mortality due to predators or starvation [97, 98, 99, 100]. Most fish, however, occur within habitats that are somewhat cooler than temperatures supporting maximum rates of growth [101]. This could be due to the need for a thermal-safety margin to survive sudden warming such as heat waves [102] or due to physiological trade-offs related to fast growth at relatively warm temperatures. For young larvae, rapid growth can lead to decrements in other fitness-related traits such as resistance to pathogens [103][91] longevity [104], energy storage [105], or locomotion [90]. In our study, sea bass larvae grew faster at 20°C but displayed poorer swimming ability than larvae reared at 15°C. The existence of a trade-off between growth and swimming ability has been reported in an increasing number of studies. Studies on fathead minnow (*Pimephales promelas*) and Atlantic silversides (*Menidia menidia*) reported a negative correlation between growth rate and locomotory performance [106, 107]. A negative correlation between growth (compensatory after food deprivation) and sprint speed was reported for juvenile sea bass [108, 109].

The mechanisms behind the trade-off between fast growth and swimming ability have been poorly investigated, although several hypotheses have been proposed. First, the trade-off is based on the principle of energy allocation [110]. Rapid growth mobilizes more aerobic capacity leaving less energy dissipating capacity for locomotion [111, 107]. Early developmental stages have a relatively low aerobic capacity with factorial metabolic scopes around 1.5 and, thus, may be especially vulnerable to energy allocation conflicts [111, 112]. Second, rapid growth in fish can influence muscle characteristics including changes in the cellular structure and composition of muscle fibers [113] leading to poor locomotor performance. This may contribute to their higher swimming performance. Third, the relationship (and potential trade-off) between growth and swimming performance may have a genetic basis and can, therefore, be population-specific [114]. It would be interesting to study additional populations that inhabit different thermal regimes to test if trade-offs differ due to local adaptation.

## Conclusion

Our results indicate that, when fed *ad libitum*, European sea bass larvae are not impacted by projected future increases in levels of *P*CO_2_ thanks to the use of physiological mechanisms allowing them to maintain growth and swimming performance. Life-time rearing at +5°C above ambient influenced larval growth, development and swimming performance. Individuals reared at warmer condition grew faster but showed reduced swimming ability at metamorphosis. While growing faster reduced the duration of the larval phase, this reduction happened at the expense of swimming performance. Although the potential for local adaptation (adaptive capacity) is not known, our findings suggest that sea bass larvae of this Atlantic population may be negatively impacted by projected climate-driven warming (but not OA) under a business-as-usual (RCP8.5) scenario. These impacts appear to be due to physiological trade-offs between growth rate and U_crit_. The physiological mechanism (i.e. limits in aerobic capacity and/or changes in energy partitioning) is unclear. The results of this and several other studies demonstrate that physiologically optimal thermal windows are stage-specific and appear more narrow (with a colder optimum) in larvae compared to juveniles. Finally, our study adds to the growing number of studies reporting no effects of OA or OAW on the swimming performance of marine fish larvae. Our review of this literature, however, also indicates that the impact of OA and/or OAW is species-specific.

## Supporting information

S1 Fig Experimental design and tanks transfer from larvae to juvenile stage. Abbreviation: dph, days post-hatch.
(TIFF)

S2 Fig Critical swimming speed (U_crit_, cm s^-1^) in juvenile European sea bass reared at A) cold condition (15°C; n = 73; 242 days post-hatch (dph)) and B) warm condition (20°C; n = 60; 233 dph). Symbols and colors indicate by *P*CO_2_ levels treatment (A, Ambient *P*CO_2_; Δ1000, ambient + 1000 μatm CO_2_).
(TIFF)

S1 Table Light intensity during larval phase. Abbreviation: dph, days post-hatch.
(PDF)

S2 Table Larval mortality (%) in the different larval rearing tanks. Abbreviations: A, Ambient *P*CO_2_; Δ500, ambient + 500 μatm CO_2_; Δ1000, ambient + 1000 μatm CO_2_; T, temperature, Rep, replicate tank.
(PDF)

S3 Table Juvenile mortality in % in the different tanks. Abbreviations: A, Ambient *P*CO_2_; Δ500, ambient + 500 μatm CO_2_; Δ1000, ambient + 1000 μatm CO_2_; T, temperature.
(PDF)

S4 Table Significance of terms for the 90% quantile regression model on the impact of water temperature and body length (BL) on the critical swimming speed (U_crit_) in European sea bass larvae. Abbreviation: DF, degrees of freedom.
(PDF)
