# Supplementary figures and images for: Combined effects of ocean acidification and temperature on larval and juvenile growth, development and swimming performance of European sea bass (Dicentrarchus labrax)
Source: PLoS One. 2019 Sep 6;14(9):e0221283. doi: 10.1371/journal.pone.0221283 (PMC6731055; doi:10.1371/journal.pone.0221283)

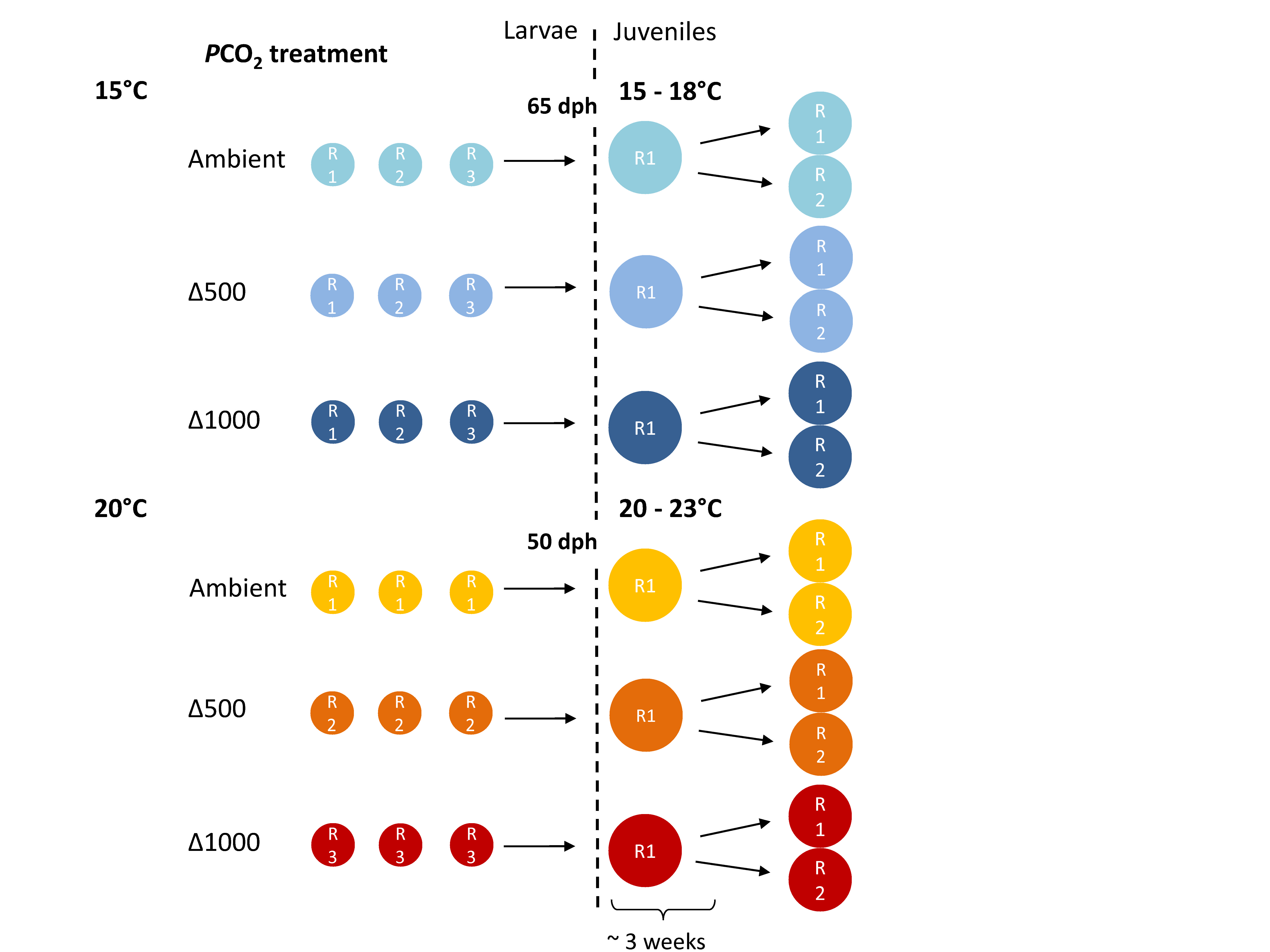

Supplement: S1 Fig — Abbreviation: dph, days post-hatch. (TIFF) [file pone.0221283.s001.tiff]

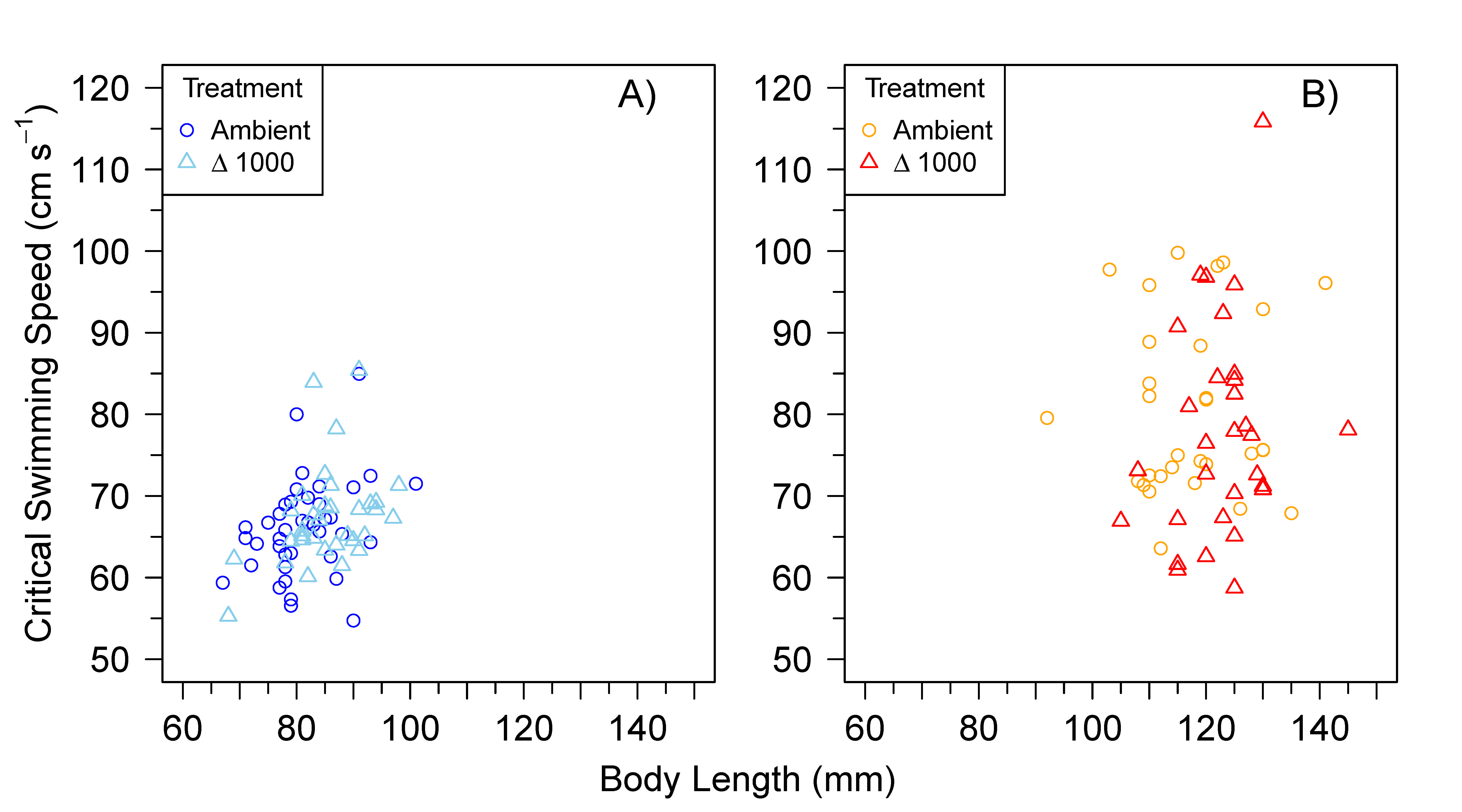

Supplement: S2 Fig — Symbols and colors indicate by PCO2 levels treatment (A, Ambient PCO2; Δ1000, ambient + 1000 μatm CO2). (TIFF) [file pone.0221283.s002.tiff]
