# Supplementary material for: Combined effects of ocean acidification and temperature on larval and juvenile growth, development and swimming performance of European sea bass (Dicentrarchus labrax)
Source: PLoS One. 2019 Sep 6;14(9):e0221283. doi: 10.1371/journal.pone.0221283 (PMC6731055; doi:10.1371/journal.pone.0221283)
Supplement: S1 Table — Abbreviation: dph, days post-hatch. (PDF) [file pone.0221283.s003.pdf]

**Age (dph)**	2	7	9	12	16	20	27	31	36	44
**Light intensity (lux)**	0	0-1	1	2	5	7	10	31	59	96
