# Supplementary material for: Combined effects of ocean acidification and temperature on larval and juvenile growth, development and swimming performance of European sea bass (Dicentrarchus labrax)
Source: PLoS One. 2019 Sep 6;14(9):e0221283. doi: 10.1371/journal.pone.0221283 (PMC6731055; doi:10.1371/journal.pone.0221283)
Supplement: S2 Table — Abbreviations: A, Ambient PCO2; Δ500, ambient + 500 μatm CO2; Δ1000, ambient + 1000 μatm CO2; T, temperature, Rep, replicate tank. (PDF) [file pone.0221283.s004.pdf]

	A			Δ500			Δ1000		
**T (°C)**	**Rep 1**	**Rep 2**	**Rep 3**	**Rep 1**	**Rep 2**	**Rep 3**	**Rep 1**	**Rep 2**	**Rep 3**
15	31.5	37.3	25.9	78.6	21.9	33.3	35.6	11.3	16.8
20	43.5	29.4	30.5	46.6	33.6	34.9	39.6	26.7	35.7
