# Supplementary material for: Combined effects of ocean acidification and temperature on larval and juvenile growth, development and swimming performance of European sea bass (Dicentrarchus labrax)
Source: PLoS One. 2019 Sep 6;14(9):e0221283. doi: 10.1371/journal.pone.0221283 (PMC6731055; doi:10.1371/journal.pone.0221283)
Supplement: S3 Table — Abbreviations: A, Ambient PCO2; Δ500, ambient + 500 μatm CO2; Δ1000, ambient + 1000 μatm CO2; T, temperature. (PDF) [file pone.0221283.s005.pdf]

T (°C)	A	Δ500	Δ1000
15	24.8	43.4	29.7
20	35.2	41.7	38.2
