# Supplementary material for: Combined effects of ocean acidification and temperature on larval and juvenile growth, development and swimming performance of European sea bass (Dicentrarchus labrax)
Source: PLoS One. 2019 Sep 6;14(9):e0221283. doi: 10.1371/journal.pone.0221283 (PMC6731055; doi:10.1371/journal.pone.0221283)
Supplement: S4 Table — Abbreviation: DF, degrees of freedom. (PDF) [file pone.0221283.s006.pdf]

	DF	Value (± SE)	p-value
Intercept	1	-27.25 (7.94)	<0.01
Body Length	1	3.19 (0.84)	<0.01
Temperature	2	1.28 (0.42)	<0.01
Body Length : Temperature	2	-0.12 (0.04)	<0.01
